# Evolving Management of Breast Cancer in the Era of Predictive Biomarkers and Precision Medicine

**DOI:** 10.3390/jpm14070719

**Published:** 2024-07-03

**Authors:** Muhammad Zubair Afzal, Linda T. Vahdat

**Affiliations:** 1 Medical Oncology, Comprehensive Breast Program, Dartmouth Cancer Center, Lebanon, NH 03755, USA; 2 Medical Oncology and Hematology (Interim), Dartmouth Cancer Center, Lebanon, NH 03755, USA; linda.t.vahdat@hitchcock.org

**Keywords:** personalized medicine, biomarkers, tumor microenvironment, immune contexture

## Abstract

Breast cancer is the most common cancer among women in the world as well as in the United States. Molecular and histological differentiation have helped clinicians optimize treatments with various therapeutics, including hormonal therapy, chemotherapy, immunotherapy, and radiation therapy. Recently, immunotherapy has become the standard of care in locally advanced triple-negative breast cancer and an option across molecular subtypes for tumors with a high tumor mutation burden. Despite the advancements in personalized medicine directing the management of localized and advanced breast cancers, the emergence of resistance to these therapies is the leading cause of death among breast cancer patients. Therefore, there is a critical need to identify and validate predictive biomarkers to direct treatment selection, identify potential responders, and detect emerging resistance to standard therapies. Areas of active scientific and clinical research include novel personalized and predictive biomarkers incorporating tumor microenvironment, tumor immune profiling, molecular characterization, and histopathological differentiation to predict response and the potential emergence of resistance.

## 1. Background on Breast Cancer

It is estimated that 310,720 new invasive breast cancer cases will be diagnosed, with 42,250 estimated breast cancer-related deaths in the year 2024 in the United States [1]. Breast cancer is heterogeneous, and its management differs significantly based on its histological, genetic, clinical, and molecular characteristics [2]. There are four major categories of breast cancer with clinical importance. These subtypes are based on the hormone receptor status expressed on the breast cancer cells (estrogen (ER) and/or progesterone (PR) receptors), the amplification/overexpression of the epidermal growth factors receptor 2 (HER2/Neu) with or without ER/PR expression, and the breast cancer cells not expressing any receptors/proteins classified as triple-negative breast cancer (TNBC) [3]. The ER/PR-expressing breast cancers are further subdivided into luminal A and luminal B tumors. Luminal A and luminal B tumors express ER and/or PR receptors, but they differ in the degree of expression and the proliferation potential measured by antigen Ki-67 (Ki-67), a proliferation marker [4]. Most breast cancers are diagnosed at an early stage, largely due to established screening guidelines and patient awareness. However, up to 10% of the patients are diagnosed at the metastatic stage [5]. These major subtypes provide the initial guidelines to cater to breast cancer-directed therapy. Endocrine therapy is the mainstay to treat ER- and/or PR-expressing breast cancers, whereas HER2/Neu-expressing breast cancers are treated with anti-HER2 monoclonal antibodies. Since TNBC lacks any target, it is conventionally treated with cytotoxic therapy with immunotherapy to cytotoxic regimens in locally advanced and metastatic TNBC [6]. It is reported that 80–90% of patients will be cured following curative intent therapy. However, approximately 20% of patients with locally advanced breast cancer patients will relapse within 10 years [4,7]. Personalized medicine encompasses the individual genomic, environmental, and lifestyle factors affecting response to standardized therapies in breast cancer patients [8]. Not every patient with similar clinical and histopathological characteristics would fare the same despite the standard therapies. Clinical response and short- and long-term clinical outcomes vary based on intrinsic tumor and unique patient characteristics that are not yet well-established. Therefore, identifying the biomarkers that define individual patient and tumor characteristics that can be used in future management strategies is paramount. There is significant development happening in breast cancer research, and most of this advancement is due to the identification of molecular factors contributing to the pathogenesis of individual breast cancers [9]. Many new therapeutic options are being developed, but the focus has shifted to developing companion diagnostic tests that could identify the most suitable therapeutics benefiting the patients [7]. This review article will focus on biomarker-driven personalized breast cancer treatment incorporating histopathological differentiation, molecular characteristics, tumor microenvironment, tumor immune profiling, the emergence of treatment resistance, and potential strategies to overcome the resistance. Changes in these biomarkers are used routinely to make treatment decisions. Biomarker monitoring can help in the early detection of progressive disease, early response detection before radiographic response, and complications emerging from the treatment [10,11]. Circulating DNA has gained traction as a biomarker to monitor response to the standard of care anti-cancer therapy. It has emerged as a more sensitive and specific biomarker with predictive and prognostic value [11]. These biomarkers can indicate a pharmacodynamic response to an intervention. These biomarkers can predict the biological activity of the drug, device, and/or other medical product. These biomarkers can identify potential harm caused by medical intervention and its effects at the cellular level [12]. Cancer therapeutics are toxic in general. The range of adverse effects varies broadly across different therapeutics and patient populations. Safety biomarkers can detect and/or predict adverse drug reactions and the degree of damage following an intervention. Based on these biomarkers, treatments could be modified, stopped, and resumed. Liver function tests and renal function tests are the simplest and most common serological biomarkers used in day-to-day clinical practice [13]. Personalized medicine, prognostic, and predictive biomarkers are pivotal in breast cancer and driving treatment strategies. ER/PR and HER2 Neu amplification are mandatory biomarkers for every breast cancer diagnosis. Androgen receptors, immuno-oncology, molecular signatures, immune modulation of the tumor microenvironment, and ctDNA-based biomarkers are emerging and are areas of active interest in breast cancer.

### Biomarkers and Precision Medicine

The biomarker indicates normal biological processes, pathological processes, and the response to an intervention. Physiological, histological, radiographic, and molecular characteristics of a tumor could define a biomarker [10]. Biomarkers can hold prognostic and predictive value. Prognostic biomarkers can identify different disease outcomes without any response assessment, whereas predictive biomarkers typically indicate a response to the treatment [10,14]. Prognostic biomarkers could identify outcomes independent of the treatment, such as relapse and progression. In breast cancer, examples of prognostic biomarkers would be the Oncotype DX and the MammaPrint, both tumor-specific gene-based markers prognosticating the risk of relapse and a potential benefit of more aggressive therapy such as chemotherapy [15]. The predictive biomarkers typically predict the potential benefit from the treatment and the potential to develop resistance to the treatment. For example, predictive biomarkers would identify potential response and resistance to standard-of-care endocrine therapy in endocrine-positive breast cancer patients. Incorporating predictive and prognostic biomarkers earlier in the treatment strategies can help determine the most appropriate patients, translating to better outcomes [16]. Diagnostic biomarkers can vary from radiographic biomarkers to molecular biomarkers. In the era of precision medicine, molecular biomarkers are taking a central stage. In oncology, the treatment is moving from tissue-/site-specific to molecular characteristic-specific treatment. For example, next-generation sequencing (NGS) has made it possible to detect mutations with targeted tumor agonistic therapies [17]. Biomarkers also have a role in serial monitoring and response to the disease’s treatment, intervention, or natural history. Prostate-specific antigen (PSA), cancer antigen 125 (CA 125), and cancer antigen 15.3 (CA 15.3) are a few examples of the biomarkers used in everyday practice (in prostate cancer, ovarian cancer, and breast cancer, respectively) to monitor response to the treatment [18].

**Figure 1:**
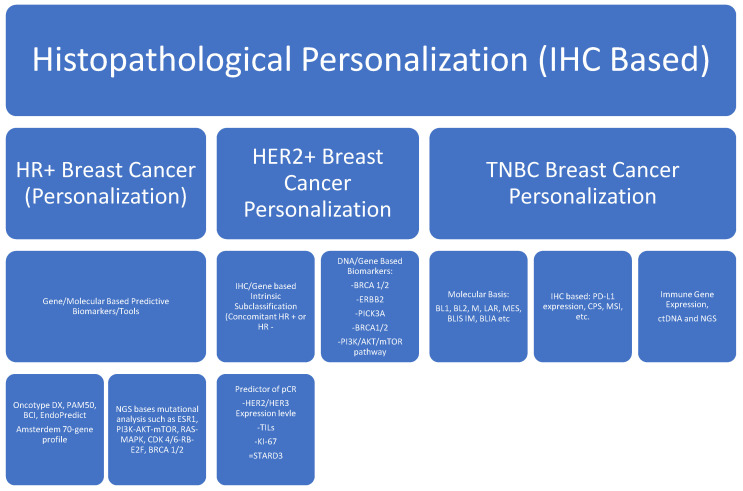
Summary of commonly used biomarkers in breast cancer.

Changes in these biomarkers are used routinely to make treatment decisions. Biomarker monitoring can help in the early detection of progressive disease, early response detection before radiographic response, and complications emerging from the treatment [10,11]. Circulating DNA has gained traction as a biomarker to monitor response to the standard of care anti-cancer therapy. It has emerged as a more sensitive and specific biomarker with predictive and prognostic value [11]. These biomarkers can indicate a pharmacodynamic response to an intervention. These biomarkers can predict the biological activity of the drug, device, and/or other medical product. These biomarkers can identify potential harm caused by the medical intervention and its effects at the cellular level [12]. Cancer therapeutics are toxic in general. The range of adverse effects varies broadly across different therapeutics and patient populations. Safety biomarkers can detect and/or predict adverse drug reactions and the degree of damage following an intervention. Based on these biomarkers, treatments could be modified, stopped, and resumed. Liver function tests and renal function tests are the simplest and most common serological biomarkers used in day-to-day clinical practice [13]. Personalized medicine, prognostic, and predictive biomarkers are pivotal in breast cancer and driving treatment strategies. ER/PR and HER2/Neu amplification are mandatory biomarkers for every breast cancer diagnosis. Androgen receptors, immuno-oncology, molecular signatures, immune modulation of the tumor microenvironment, and ctDNA-based biomarkers are emerging areas of active interest in breast cancer (Figure 1).

Hormone positive, HR: human epidermal growth factor receptor-2, HER2: triple-negative breast cancer, TNBC: antigen Ki-67, Ki-67: estrogen receptor gene-1, ESR-1: rat sarcoma protein, RAS: mitogen-activated protein kinases, MAPK: phosphoinositide-3-kinase, PI3K: protein kinase B, Akt: the mechanistic target of rapamycin, mTOR: cyclin-dependent kinase 4/6 CDK 4/6: retinoblastoma, RB: early region 2 binding factor (E2F), IM: immunomodulatory, BLIA: basal-like immune activated, M: mesenchymal, MSL: mesenchymal stem-like, BL: basal-like, LAR: luminal androgen receptor, TILs: tumor-infiltrating leukocytes, CPS: combined positive score, PD-L1: programmed death ligand-1. 

## 2. Personalization Based on Histopathological and Clinical Parameters

The management of breast cancer begins with histological and clinical differentiation. Histological differentiation characterizing luminal A, Lumina B, HER2 amplification, and the TNBC subtype determines the type of therapy to be offered [3]. Luminal A and luminal B tumors express ER and/or PR receptors, but they differ in the degree of expression and the proliferation potential that is measured by antigen Ki-67 (Ki-67) [4].

### 2.1. Personalization in Endocrine-Positive Breast Cancer

Approximately 70–75% of the patients with breast cancer patients are ER/PR positive [19]. The immunohistochemical (IHC) evaluation of breast cancer to determine ER/PR status is the gold standard and predicts the response to the endocrine therapy. Endocrine therapy in adjuvant settings decreases local and distant relapse rates and also provides secondary prevention for second breast cancer [20]. However, patients with high-risk diseases may require additional systemic therapy like chemotherapy. Several predictive tools are developed to determine the risk of recurrence and the potential benefit of chemotherapy. The most widely used marker is Oncotype DX 21-gene recurrence score, which has been validated by TAILORx and RxPONDER clinical trials; it is also the most recommended in National Comprehensive Cancer Network (NCCN) guidelines. But the Breast Cancer Index, Predictor Analysis of Microarray 50 (PAM50), EndoPredict, and the Amsterdam 70-gene profile are a few other tools available to determine the risk of recurrence in endocrine-positive breast cancers [21]. 

#### 2.1.1. Endocrine Resistance

Approximately 20–40% of the patients on endocrine therapy will eventually develop endocrine resistance [22]. Primary endocrine resistance is a relapse within 2 years of adjuvant treatment or 6 months of first-line endocrine therapy in advanced/metastatic breast cancer. Secondary endocrine resistance is the relapse after 2 years of adjuvant endocrine therapy or progression following 6 months of endocrine therapy in advanced/metastatic breast cancer [23].

There are several mechanisms of endocrine resistance in breast cancer. ER expression in individual breast cancer patients is a dynamic process, and in approximately 10–20% of the cases, ER expression may be lost or changed, resulting in a lack of endocrine therapy responsiveness and the emergence of resistance [23,24]. Other proposed mechanisms of endocrine resistance included genomic and epigenetic, estrogen receptor gene-1 (ESR-1) alterations, truncated ER-isoform expression, estrogen/progesterone receptors pathway aberrations resulting from ER expression dysregulation, post-translational modification, increased receptor tyrosinase kinase signaling, altered cell cycle regulation, genetic and epigenetic factors affecting uptake, metabolism, and cellular responses of endocrine agents [25,26].

ESR1 mutations are common causes of acquired resistance to endocrine therapy. ESR1 mutations account for 20–40% of the resistance in metastatic breast cancer cases receiving endocrine therapy. The prevalence of the ESR1 alteration also varies by the disease settings [27,28]. ESR1 prevalence is merely 4–5% in adjuvant endocrine therapy settings and 1.5–7% in neoadjuvant settings [28,29]. The de novo ESR1 mutation is sporadic and is only seen in 0.5–1% of metastatic breast cancer patients without prior endocrine therapy [30].

Multiple ESR1 mutations were discovered with genomic sequencing of the metastatic breast cancer. The most common mutations are D538G, Y537S, Y537N, Y537C, and E380, which occur at hot spots in the ligand-binding domain of ERa. These mutations are associated with the aggressive biology [31,32]. 

**Figure 2:**
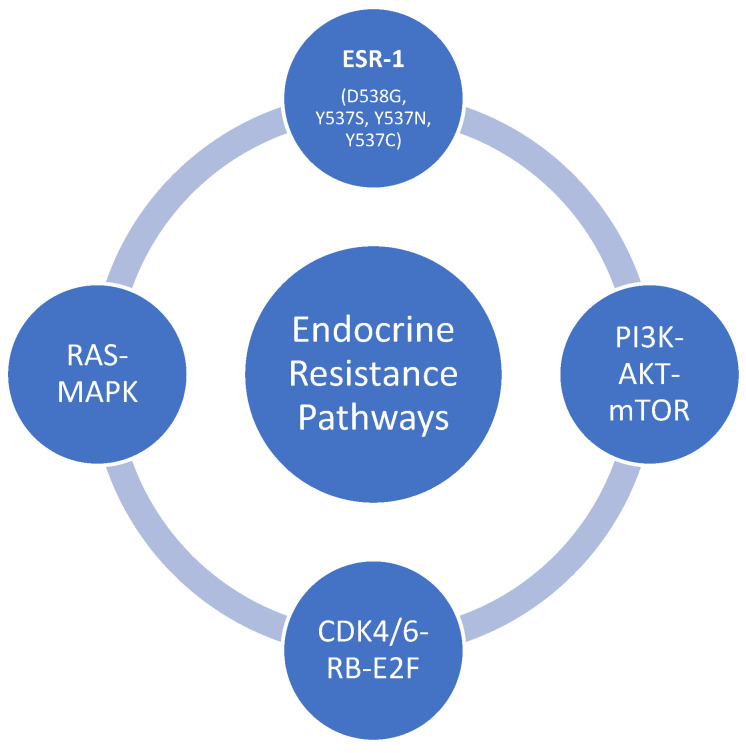
Pathways involved in the endocrine resistance mechanism. Estrogen receptor gene-1, ESR-1; rat sarcoma protein, RAS; mitogen-activated protein kinase, MAPK; phosphoinositide-3-kinase, PI3K; protein kinase B, Akt; mechanistic target of rapamycin, mTOR; cyclin-dependent kinase 4/6, CDK 4/6; retinoblastoma, RB; early region 2 binding factor, E2F.

Other pathways that may be implicated in the development of resistance to endocrine therapy include PI3K-AKT-mTOR, RAS-MAPK, and CDK4/6-RB-E2F pathways. Tyrosine kinase is the intracellular domain of cell membrane-bound receptors, receptor tyrosine kinases (RTK). There are various RTKs, such as epidermal growth factor receptors, insulin-like growth factors receptors, fibroblast growth factor receptors, and vascular growth factors receptors. These RTKs are activated by ligand binding, such as hormones, cytokines, and growth factors [33,34]. The bindings of these ligands to RTKs activate intracellular signal transduction pathways such as mitogen-activated protein kinases (MAPKs) and phosphoinositide 3-kinase (P13K)/AKT pathways [35]. These pathways could be responsible for the transcriptional activities of the estrogen receptors, and the alteration of these pathways could also lead to potential resistance to endocrine therapy, especially in metastatic breast cancer [36]. The mammalian target of rapamycin complex (mTOR) forms an essential effector of the PI3K/AKT pathway that provides positive feedback to the PI3K/AKT pathway and results in regular tumor growth, survival, motility, metabolism and eventually evading the effect of endocrine therapy [37,38]. The cyclin D/cyclin dependent kinases (CDK) 4/6/retinoblastoma (Rb) pathways regulate the G1-S checkpoint in the cell cycle and control the progression of the cancer cells. Sustained activation of CDK 4/6 and inactivation of Rb via phosphorylation by CDK4/6 leads to cell cycle activation and proliferation, decreasing efficacy or non-responsiveness to endocrine therapy [39,40]. Resistance from ESR1 mutation can emerge while on CDK 4/6 inhibitors (CDK 4/6i) in combination with endocrine therapy. In a randomized trial on switching to fulvestrant and Palbociclib versus no switch in metastatic breast cancer with rising ESR1 mutation while on aromatase inhibitors and palbociclib, there was a 27% rise in ESR1 mutation based on ctDNA analysis (Figure 2) [41].

#### 2.1.2. Overcoming the Resistance to Endocrine Therapy

Despite the emergence of endocrine resistance, endocrine-positive breast cancer treatment still depends on ER signaling. New generations of novel anti-estrogen therapies are designed to curb various resistance mechanisms. These therapies include the existing classes of anti-estrogen treatments, such as selective estrogen receptor modulators (SERMs) and selective estrogen receptor degraders (SERDs). Other novel anti-estrogen drugs include selective estrogen receptors covalent antagonists (SERCAs), proteolysis-targeting chimeric (PROTACs) targeting estrogen receptors, and complete estrogen receptor antagonists (CERNAs) [42]. Some next-generation anti-estrogen therapies to overcome endocrine resistance are outlined below (Table 1).

a. Elacestrant.

Elacestrant is an oral SERM/SERD hybrid agent approved by the FDA in January 2023 based on EMERALD (NCT03778931), a randomized open-label phase III trial. This trial included patients with ESR1 mutation and ESR1*wt*. The primary efficacy outcome measure was progression-free survival (PFS). Among 228 patients with ESR1 mutation, the median PFS was 3.8 months in the elacestrant arm and 1.9 months in the fulvestrant or aromatase inhibitor arm (HR = 0.55, 95% CI: 0.39–0.77]. Among patients without ESR1 mutation, HR was 0.86 (95% CI: 0.63–1.19). Therefore, the FDA only approved elacestrant in patients with ESR1 mutation [43,44]. Elacestrant is currently being studied in neoadjuvant settings based on Ki-67 dynamics (NCT04797728) and in combination with abemaciclib in patients with brain metastasis (NCT04791384) [45,46]. Trials incorporating elacestrant in combination with samuraciclib and various other combinations in metastatic breast cancer as well as in CDK4/6i naïve metastatic breast cancer patients (NCT05963997, NCT05563220, NCT05596409 respectively) are actively recruiting [47,48,49].

b. Camizestrant.

Camizestrant is an oral SERD that suppresses tumor growth in patients with ESR1 mutation. In a phase I SERENA-1 trial (NCT03616586), in a heavily pretreated population, camizestrant demonstrated clinical activity as a monotherapy with ORR of 10%, CBR of 35.3% across all dose levels, and CBR of 53.3% with median PFS of 11.1 months at 75 mg dose [50]. The dose expansion cohort of camizestrant, 75 mg, in combination with palbociclib, revealed an ORR of 6.3% and a CBR of 50% [51]. There are further ongoing trials on camizestrant in advanced endocrine-positive breast cancer. SERENA-2 (NCT04214288) is a randomized phase II trial that compares the efficacy and safety of camizestrant in comparison with fulvestrant at three dose levels after at least one endocrine therapy progression [52]. SERENA-4 (NCT04711252) compares camizestrant in combination with palbociclib compared to AI and palbociclib [53]. SERENA-6 (NCT04964934) compares AI and CDK 4/6 inhibitors with camizestrant instead of AI, continuing the same CDK 4/6i once ESR1 mutation is detected after 6 weeks of AI plus CDK 4/6i therapy without radiographic progression [54]. SERENA-3 (NCT04588298) is a window-of-opportunity trial involving postmenopausal women (in a neoadjuvant setting) with ER-positive localized breast cancer, receiving 75 mg to 150 mg of camizestrant to evaluate the effect of this drug on ER expression [55].

c. Imlumestrant.

Imlumestrant is an oral SERD demonstrating potent inhibition of ESR1*wt* and mutant breast cancer cells. In a phase I/II EMBER-1 trial, imlunestrant was combined with alpelisib, abemaciclib, everolimus, and trastuzumab +/− abemaciclib in premenopausal and post-menopausal women with breast cancer and endometrial cancer. In the trial, ctDNA-based ESR1 analysis was conducted, and this combination demonstrated a 73% clearance or decline of ESR1 ctDNA levels. The median PFS was 6.5 months in the imlunestrant cohort compared to 4.3 months [56]. EMBER-3 (NCT04975308) is a phase III randomized study investigating imlunestrant as a monotherapy or in combination with abemaciclib in patients with previously treated endocrine-positive breast cancer [57]. Imlunestrant is also being studied in neoadjuvant and adjuvant settings (EMBER-2, NCT04647487, and EMBER-4) [56,58].

d. Lasofoxifene.

SERMs display estrogen receptor agonist or antagonist activity depending on the target cells. Agonist activity relies on the activating function domain 1 (AF1) through Pi3K, MAPK, and mTOR pathways. The antagonist activity is relayed by inhibiting estrogen receptors’ activating function domain 2 (AF2). Tamoxifen is the first SERM and most used in adjuvant and metastatic settings. Raloxifene is another SERM used in breast cancer prevention strategies. 

Lasofoxifene is a next-generation non-steroidal SERM. Among patients with ESR1 mutation, lasofoxifene has been shown to inhibit tumor growth compared to fulvestrant. Lasofoxifene was compared with fulvestrant in the ELAINE trial (NCT03781063) among pre- and postmenopausal patients with ESR1 mutation. These patients had previously received CDK 4/6 inhibitors. Lasofoxifene demonstrated improved median PFS compared to fulvestrant (6.04 vs. 4.04 months, HR 0.69, *p* = 0.13) [59]. ELAINE II (NCT04432454) is a phase II randomized trial evaluating lasofoxifene in combination with abemaciclib. This trial is still ongoing [58].

e. Rintodestrant.

Rintodestrant is a novel oral SERD that has demonstrated activity in ESR1 mutant tumors. In a dose expansion of the phase I trial, rintodestrant demonstrated ORR of 5% and CBR of 30% in pre- and postmenopausal women. The activity was observed regardless of ESR1 and PIK3CA status. This trial further assessed rintodestrant in combination with palbociclib in recurrent settings without prior CDK 4/6i but endocrine therapy exposure. Initial data have shown an ORR of 5% and a CBR of 60% [60,61].

f. SERDS and other novel agents in ESR1 mutant breast cancers.

Borestrant is boronic acid-modified orally bioavailable SERD demonstrating the downregulation of estrogen receptors in endocrine-resistant breast cancer cells compared to fulvestrant. ENZENO (NCT04669587) is an ongoing trial evaluating the safety and tolerability of borestrant as a single agent and in combination with palbociclib in endocrine-positive advanced/metastatic breast cancer patients [62]. D-0502 is another oral SERD being studied in a phase I trial (NCT03471663) as monotherapy and in combination with palbociclib in postmenopausal and pre-menopausal women on ovarian suppression with advanced endocrine-positive breast cancer patients. Preliminary results showed improvement in ORR and CBR in both monotherapy and combination cohorts [63].

ZN-c5, another orally bioavailable SERD, is being evaluated in a phase I/II trial (NCT03560531) as monotherapy or in combination with palbociclib in pre- and postmenopausal women with advanced ER-positive breast cancer. This compound has shown an ORR of 5% and a CBR of 38%. Phase II of this trial is in progress, testing the combination of this compound with palbociclib [64,65]. 

SERCAs inactivate estrogen receptors by interacting with unique cysteine residue specific to estrogen receptors [66]. In the phase I/II trial evaluating compound H3B-6545 in pre- and postmenopausal women with advanced endocrine-positive breast cancer, the ORR was 16.4% and CBR was 39.7% with a median PFS of 3.8 months. These patients had previously received at least three lines of therapy, including CDK 4/6i [67]. H3B-6545 is also being tested in combination with palbociclib in patients with endocrine-positive advanced breast cancer who had previously received two lines of therapy (NCT04288089) [68]. 

CERANs block activation domains AF1 (activated by mTOR, P13K, MAPK pathways) and AF2 (activated by estrogen ligand), leading to the depression of gene transcription and cell proliferation. CERNs block AF1 and AF2 in contrast to SERMs, which block only AF2 but show agonist activity via AF1 [69]. OP-1250 is a bioavailable CERAN demonstrated in ESR1 mutant breast cancers. NCT04505826 is the first-in-human study evaluating OP-1250. Dose expansion, during phase 2 of this trial, showed an ORR of 9% and CBR of 21% with drug tolerability. At the recommended phase 2 trial dose, the ORR was 18%, and CBR was 38% [70]. 

Although the estrogen receptor remains the primary predictive biomarker of response to endocrine therapy, the emergence of resistance is a significant challenge. Several strategies, as outlined above, are being investigated, with elacestrant being the latest addition used to treat resistant endocrine-positive breast cancer. More promising agents are at various stages of development [Table 1].

**Table 1:** Overcoming the resistance to endocrine therapy (therapeutics and ongoing trials).

Agent	Class	Clinical Trials	Patient Population	Endpoints	Salient Results	Status
Elacestrant [43,44]	Oral SERM/ SERD	EMERALD (NCT03778931) Randomized P-III	Patients with ESR1 mut. and EST_wt_	PFS	ESR1 mut. = mPFS 3.8 vs. 1.9 months [HR = 0.55, 95% CI: 0.39–0.77] ESR1wt HR 0.86 (95% CI: 0.63–1.19)	Yes, only in patients with ESR1 mutation
Elacestrant + Abemaciclib [46]	Oral SERM/ SERD + CDK 4/6i	NCT04791384 (P-Ib and II)	Breast cancer with brain metastasis	Safety and tolerability ORR CBR		Ongoing
Elacestrant + Samuraciclib [47]	Oral SERM/ SERD + CDK7	NCT05963997 (P-Ib/2)	Locally advanced or metastatic patients HR-positive, HER2-unamplified	Safety and Tolerability, PFS, ORR, CBR, DOR		Ongoing
Elacestrant + everolimus, alpelisib, palbociclib and ribociclib [48]	Oral SERM/ SERD + CDK 4/6i or mTOR inhibitor or PIK3CAi	NCT05563220 (P-Ib/2)	Locally advanced or metastatic patients HR-positive, HER2-unamplified	Safety and tolerability, PFS, ORR, CBR, DOR, OS		Ongoing
Elacestrant [49]	Oral SERM/SERD	NCT05596409 (early P-II)	Locally advanced or metastatic patients HR-positive, HER2-unamplified (CDK 4/6i naïve)	PFS, OS, CBR, DOR		Ongoing
Camizestrant	SERD	SERENA-1 (NCT03616586) P-1	Heavily pre-treated patients with ESR-1 mut.	ORR, CBR, PFS	ORR 10% CBR 35.3% across all dose levels. CBR 53.3% a dmPFS for 11.1 months at 75 mg dose	Ongoing
Camizestrant + Palbociclib [51]	SERD + CDK 4/6i	SERENA-1 (NCT03616586) Dose expansion cohort	Heavily pre-treated patients with ESR-1 mut.	ORR CBR	ORR 6.3% CBR 50%	Ongoing
Camizestrant vs. Fulvestrant [52]	SERD	SERENA-2 (NCT04214288) P-II	Heavily pre-treated patients	PFS, ORR, DOR, CBR		Ongoing
Camizestrant + Palbociclib vs. AI + Palbociclib [53]	SERD + CDK 4/6 i	SERENA-4 (NCT04711252) P-III	Denovo stage IV or locally advanced early-stage breast cancer	PFS, OS, CBR, secondary PFS		Ongoing
AI + CDK 4/6i vs. AI + CDK 4/6i + Camizestrant [54]	SERD + CDK 4/6 i	SERENA-6 (NCT04964934)	Metastatic or locally advanced Ca breast with ESR1 mut. emergence before radiographic progression	PFS1, PFS2, OS, ORR, CBR, QOL		Ongoing
Imlunestrant + alpelisib, abemaciclib, everolimus, trastuzumab +/− abemaciclib [56]	SERD + CDK 4/6i, anti-HER2 mAb, mTORi	EMBER-I P-I/II (NCT04647487)	Metastatic breast and endometrial cancer	Safety and tolerability, ESR-1 clearance, mPFS	73% clearance or ctDNA with ESR-1 mutation, mPFS 6.5 vs. 4.3 months.	Ongoing
Imlunestrant vs. Imlunestrant + abemaciclib [57]	SERD + CDK 4/6i,	EMBER-3 3 (NCT04975308), P-III	Previously treated endocrine-positive tumors	PFS, OS, ORR, CBR		Ongoing
Lasofoxifene vs. Fulvestrant [59]	SERM	ELAINE-I trial (NCT03781063), P-II	Previously treated endocrine-positive tumors with ESR-1 mut.	PFS; safety and tolerability	mPFS 5.6 vs. 3.7 months, *p =* 0.138, CBR 36.5% vs. 21.6%; *p* = 0.117, ORR 13.2% vs. 2.9%; *p* = 0.124	Ongoing
Lasofoxifene + Abemaciclib [58]	SERM + CDK 4/6i	ELAINE-II (NCT04432454), P-II	Previously treated endocrine-positive tumors with ESR-1 mut.	Safety and tolerability, PFS, CBR, ORR, DOR		Ongoing
Rintodestrant + Palbociclib [60,61]	SERD + CDK 4/6i	P-1/P-II	Previously treated endocrine-positive tumors with ESR-1 mut. (without prior CDK 4/6 exposure)	Safety and tolerability, ORR, CBR	ORR 5%, CBR 60%	Ongoing
Borestrant (monotherapy) Or Borestrant + Palbociclib [62]	SERD/SERD + CDK4/6i	P-I and P-II (NCT04669587)	Metastatic or locally advanced Ca Breast	Recommended dose, response as monotherapy, response in combination, ORR, CBR		Ongoing
D-0502 (monotherapy) Or D-0502 + Palbociclib [63]	SERD/SERD + CDK4/6i	P-I (NCT03471663)	Metastatic or locally advanced breast cancer	MTD, DLT, ORR, PFS	Combination was better	Ongoing
ZN-c5 Or ZN-c5 + Palbociclib [64,65]	SERD/SERD + CDK 4/6i	P-I/II (NCT03560531)	Pre- and postmenopausal women with advanced ER-positive breast cancer	MTD, RP2D; safety and tolerability. ORR, CBR	Monotherapy showed ORR of 5% and CBR of 38%	P-II is still ongoing
H3B-6545	SERCA	P-I/II	Pre- and postmenopausal women with advanced ER-positive breast cancer (patient received at least 3 previous lines of therapy), including CDK 4/6i	MTD, ORR, CBR, PFS	ORR = 16.6% CBR 39.7% mPFS = 3.8 months	
H3B-6545 + Palbociclib [67,68]	SERCA + CDK 4/6i	P-I (NCT04288089)	Pre- and postmenopausal women with advanced ER-positive breast cancer (patient received at least 3 previous lines of therapy)	MTD, ORR, CBR, DOR		Ongoing
OP-1250 [70]	CERAN	P-I and P-II (NCT04505826)	Pre- and postmenopausal women with advanced ER-positive breast cancer with ESR-1 mut	DLT, MTD, ORR, CBR	P-I “ORR = 18%, CBR 38%” P-II ORR = 18%, CBR 38%	

SERD: selective estrogen receptor degrader, SERM: selective estrogen receptor modulator, ESR1: estrogen receptor 1, ESR1wt: estrogen receptor 1 wild-type, mPFS: median progression-free survival, CKD 4/6i: cyclin-dependent kinase-1 inhibitor, HR: hormone receptor, ORR: objective response rate, CBR: clinical benefit rate, DOR: duration of response, OS: overall survival, QOL: quality of life, MTOR: the mammalian target of rapamycin, PIK3CA: phosphatidylinositol-4,5-bisphosphate 3-Kinase, mAb: monoclonal antibody, MTD: maximum tolerated dose, DLT: dose-limiting toxicity, RP2D: recommended phase 2 dose, SERCA: sarcoplasmic/endoplasmic reticulum Ca^2+^-ATPase, CERAN: complete estrogen receptor antagonist.

## 3. Personalization in Human Epidermal Growth Factor Receptos-2 (HER2)-Amplified Breast Cancer

HER2 is amplified in about 15–20% of breast cancers [71]. HER2-directed therapies are the cornerstone of the management of HER2-amplified breast cancers. This has significantly improved cancer-related outcomes with median survival over 50 months in HER2-amplified cancer patients [72]. HER2-positive breast cancer is a heterogeneous disease. Concomitant endocrine positive status (ER+ and ER−ve HER2-amplified tumor), intrinsic subtypes, ERBB2 mRNA levels, BRCA1/BRCA2 mutations, ERBB2 mutation/amplification, PIK3CA mutation, and immune microenvironments, such as TILs, PD-L1, and FcyR alleles, all contribute to the heterogeneity of HER2-amplified breast cancers [73,74]. 

### 3.1. Level of HER2 and HER3 Expression

Although HER2 expression is indicated by amplified or unamplified status, the level of mRNA translating ERBB2 varies proportionately from IHC-0 to IHC-3 for HER2-expressing tumor cells [75]. The level of HER2 expression can be prognostic. Per the CLEOPATRA trial, low HER2 expression indicated poor median PFS compared to higher HER2 expression (HR 0.77, 95% CI 0.63–0.93, *p* = 0.008). In the EMILIA trial, in patients with high ERBB2 mRNA, the median PFS was 10.6 months compared to 8.2 months in patients with low ERBB2 mRNA treated with T-DM1 [76]. In TH3RESA, the median PFS was 7.2 months vs. 5.5 months with T-DM1 in patients with high ERBB2 mRNA expression [76]. MARIANNE trial showed similar outcomes in patients with high ERBB2 mRNA levels treated with T-DM1 (median PFS 18.6 months vs. 10.2 months). In the same trial, patients with IHC 3+ HER2 had a median PFS of 14.6 months vs. 7.3 months in patients with IHC 2+ HER2 [77]. In all these trials, the absolute difference in median PFS was lower among patients with lower HER2 expression or ERBB2 mRNA levels. 

Trastuzumab deruxtecan has demonstrated clinical activity in patients with low HER2 expression (traditionally characterized as HER2-unamplified). Among patients with low HER2 expression, the response rate to trastuzumab deruxtecan was 37% in heavily pretreated metastatic breast cancer patients. The median PFS was 11.1 months, and the duration of response was 10.4 months [78]. 

Intratumoral heterogeneity of HER2 can also determine treatment-related outcomes. Intratumoral heterogeneity can present as a clustered type with different HER2 levels within the same tumor, a mosaic type with diffuse and variable HER2 expression among individual cancer cells, and a scattered type with HER2-amplified cells scattered within an otherwise HER2-unamplified tumor [74]. In the KRISTINE trial, which evaluated neoadjuvant T-DM1 and pertuzumab compared to trastuzumab, pertuzumab, docetaxel, and carboplatin, patients experiencing locoregional progression before surgery showed a higher heterogeneity in tumor cell populations, with variable HER2 IHC expression in 80% of the tumor cells. In contrast, among the patients with no locoregional progression, 85% had homogeneous HER2 expression [79]. Similarly, post hoc analysis of the MATIANNE trial also evaluated the impact of HER2 heterogeneity on the treatment-related outcomes in patients with heterogeneous HER2 expression, showing poor responses to T-DM1. The median PFS was 14.7 months in HER2 homogeneous tumors compared to <10 months in more heterogeneous tumors [76]. Despite these reports, reporting the HER2 heterogeneity is not standard, and the treatment has not been modified in standard clinical practice. 

HER3 is another TKR, along with the epidermal growth factor receptor (EGFR) and HER2, which plays a vital role in cellular proliferation [80]. HER2-HER3 together provides the most active signal for cellular proliferation. HER3 has also been shown to play an essential role in the development of resistance to anti-HER2 therapy [81,82]. However, in multiple clinical trials, the association between different levels of HER3 expression and treatment-related outcomes has produced variable results [76,77]. 

Despite variable evidence and unclear roles in tumorigenesis, anti-HER3-directed therapies are at various stages of development. MCLA-128 is a bispecific antibody targeting HER2 and HER3 receptors and is being evaluated in a phase II trial (NCT03321981). This antibody also potentially blocks the HER3 ligand-induced receptor demineralization. This phase II trial is planned for HER2 low, ER-positive breast cancer patients with advanced disease after progression on CDK 4/6i. The preliminary data have shown a disease control rate of 45% [83]. Patritumab is a monoclonal antibody directed against HER3. In a phase I study, in combination with paclitaxel and trastuzumab, patritumab demonstrated affordable toxicity [84]. 

Patritumab deruxtecan is a HER3-directed antibody-drug conjugate being studied in phase I/II trials in patients with metastatic breast cancer expressing HER3. The preliminary results showed promising activity in ER+ve/HER2−ve, triple negative, and HER2+ breast cancers [85]. Lumretuzumab and seribantumab are other HER3-directed antibodies that have shown significant toxicities in preclinical trials. No efficacy data about these agents are available yet [86,87]. Although HER3-directed therapies may not be ready for ‘prime time’ yet, they could be promising therapeutic targets and prognostic biomarkers for breast cancer as more data become available. 

### 3.2. DNA and Gene-Based Biomarkers in HER2-Positive Tumors

**Figure 3:**
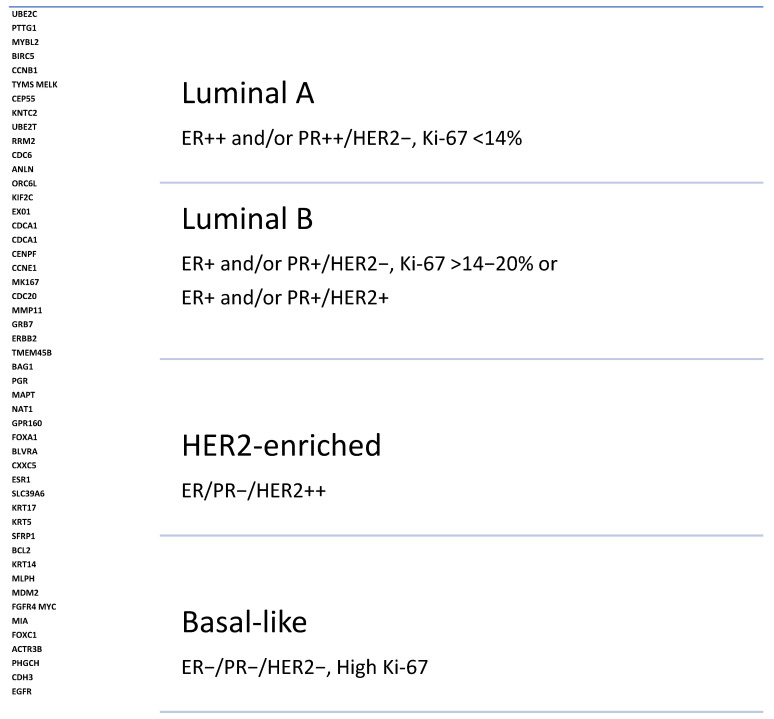
PAM50 genes and intrinsic breast cancer subtypes based on these genes. Estrogen receptor, ER; progesterone receptor, PR; human epidermal growth factor receptor-2, HER2; antigen Ki-67, Ki-67.

HER2-amplified breast cancers can present as any of the four intrinsic subtypes of breast cancer, depending on the co-expression of endocrine receptors. These subtypes are HER2-enriched, basal-like, luminal A, and luminal B [88]. These intrinsic subtypes of HER2-amplified breast cancer can determine treatment-related outcomes and can potentially be used as prognostic biomarkers [89]. The PAM50-based breast cancer subtype in endocrine-positive and HER2-amplified tumors demonstrated that patients with luminal A cancer experienced longer median PFS. In the luminal A cohort, the median PFS was 11 months, it was 5.6 months in the luminal B cohort, 4.4 months for HER2-amplified, and 3.6 months in basal-like metastatic breast cancers [90]. These studies suggest the utility of gene-based intrinsic breast cancer subtyping and endocrine receptor expression as dual biomarkers in localized and metastatic breast cancer patients (Figure 3).

The analysis of germline and somatic DNA mutation in solid tumors, including breast cancer, has become a standard of care in advanced settings and is now increasingly being explored in early-stage settings. This DNA analysis not only provides additional biomarkers for tumor-directed therapies but also holds the potential to be used as a predictive biomarker. However, in HER2-amplified breast cancers, the role of DNA sequencing using NGS is still in the investigational phase, and there are no FDA-approved DNA mutation-based targeted therapies yet. This ongoing research underscores the evolving landscape of breast cancer treatment. 

ERBB2 mutation is mainly seen in HER2-amplified breast cancers, with an overall incidence of about 3% in all breast cancers [91]. ERBB2 has oncogenic potential and could be a target for HER2-directed therapies. These therapies are tyrosine kinase inhibitors, and a few trials are underway to target ERBB2 tyrosinase kinases in HER2-amplified tumors (NCT02544997 and NCT03412382) [74].

BRCA 1/2 germline mutations are common genetic alterations in breast cancer, but there are no FDA-approved BRCA 1/2-directed therapies in HER2-amplified breast cancers, as HER2-amplified breast cancer with BRCA mutations were typically excluded from the trials. BRCA 1 is the most seen in TNBC, and BRCA 2 is the most seen in endocrine-positive breast cancers. The incidence of BRCA 1/2 in HER2-amplified breast cancers is ~4% [92,93]. 

PIK3CA is a common gene alteration seen in breast cancers. PIK3CA mutations are seen on exons 9 and 20, with HER2-amplified tumors exhibiting PIK3CA on exon 9 [89,94,95]. The PIK3CA mutation is associated with poor response to anti-HER2 therapy in neoadjuvant and metastatic settings [94,96]. This has stemmed from the hypothesis that the PIK3CA mutation is associated with potential resistance to anti-HER2 therapy [97]. PIK3CA-targeted therapies are commonly employed in endocrine-positive breast cancer patients targeting the PI3K/AKT/mTOR pathway [98,99]. However, the role of this pathway in HER2-amplified breast cancers is still under investigation. Multiple studies have evaluated the role of PI3Ki in HER2-amplified breast cancers. Buparlisib is a PI3KCAi that has been trialed in HER2-positive breast cancer in phase Ib and phase II studies in combination with lapatinib and trastuzumab, respectively. Buparlisib, in combination with lapatinib, demonstrated an ORR of 4% and CBR of 29% [100], and in combination with trastuzumab, the ORR was 10% [101]. Pilarasib was also studied in phase I/II trials in combination with trastuzumab vs. trastuzumab and paclitaxel. The results were disappointing, with an ORR of 0% for trastuzumab and 20% for trastuzumab and paclitaxel [102]. Taselisib was investigated in combination with T-DM1 in a phase Ib trial, which demonstrated an ORR of 33% with a median PFS of 7.6 months [103]. Alpelisib, in combination with T-DM1 in the phase I trial, demonstrated an ORR of 43% with a median PFS of 8.1 months [104]. In another phase I trial, alpelisib demonstrated stable disease in 83% of the breast cancer patients when used in combination with trastuzumab + LJM716. However, the total number of patients was only 6 [105]. IPATHER is an ongoing phase Ib trial evaluating the combination of PIK3CAi ipatasertib in combination with pertuzumab + trastuzumab in advanced HER2-positive PI3KCA mutant breast cancer (NCT04253561) [106]. The role of gene testing and NGS in evaluating DNA mutation is evolving in HER2-amplified breast cancer, and there is potential for NGS-based biomarker utility in HER2-amplified breast cancer patients. 

### 3.3. Biomarkers for Predicting Pathological Response to HER2-Directed Therapy

Neoadjuvant therapy is a standard of care for HER2-amplified locally advanced breast cancer [107]. Anti-HER2 therapy in neoadjuvant settings can achieve pCR in over 60% of cases, especially with a dual blockade by two anti-HER2 monoclonal antibodies [108,109]. Although achieving pCR is associated with improved long-term outcomes, many patients do not achieve pCR. Several factors can indicate the probability of pCR; however, they are not routinely used in clinical practice [110]. One of the most studied biomarkers predicting the response to neoadjuvant therapy is the level of HER2 expression. High ERBB2 mRNA and associated proteins activate the EGFR-HER2 signaling pathway, producing high pCR [111,112]. The P1K3CA pathway is also essential in HER2-amplified breast cancer. PIK3CA is present in the HER2 downstream signaling pathway [113]. PIK3CA is regulated by PTEN expression, which is present further downstream in the pathway. Activation of PIK3CA mutation and loss of PTEN lead to aggravated PIK3CA signaling, resulting in aggressive behavior [114]. The presence of PIK3CA mutation and loss of PTEN are associated with poor response to anti-HER2 therapy. However, as reported above, adding PIK3CA inhibitors has resulted in a meager response in HER2-amplified cancers [100,101,102,115]. 

Tumor-infiltrating leukocytes (TILs) indicate immunogenic hot tumors with recruitment of immune modulators and antigen-presenting cells, regulatory T-cells that can result in increasing anti-tumor activity and indicate increased response to neoadjuvant therapy [116,117]. In the GeparSixto trial, 20% of patients were classified as having lymphocyte-predominant breast cancer. These patients had a higher rate of pCR than those with lower lymphocyte infiltration levels (64% vs. 27%) [118]. In the NeoALTTO trial, in patients with greater than 5% TILs, pCR was higher compared to the patients with a lower percentage of TILs [119]. High Ki-67 is also reported to be associated with a higher response to neoadjuvant therapy [120]. Molecular crosstalk between HER2 amplification and hormone receptors also leads to poor response to neoadjuvant treatment. This crosstalk supports the hypothesis that estrogen binding to cytoplasmic estrogen receptors activates HER2 blockage, bypassing the signaling pathway [121,122]. In almost all the trials on neoadjuvant therapy in HER2-amplified breast cancer, pCR was significantly lower in hormone-positive patients compared to hormone receptor-negative patients [123,124]. 

StAR-related lipid transfer domain-3 (STARD3) is co-amplified and co-expressed with HER2 in breast cancer [125]. Studies have shown that STARD3 silencing is associated with restricted cellular growth [126]. HER2-amplified breast cancers have a particular tendency toward STARD3 expression, and STARD3 co-expression is implicated toward pCR in HER2-amplified breast cancers. Higher STARD3 expression may be associated with higher sensitivity and pCR to the anit-HER2-directed therapy [127,128]. 

Although HER2 amplification and its magnitude are major predictors of response to anti-HER2 therapy, other potential markers also play a role in predicting response and prognosis following anti-HER2 therapy. Future studies and predictive models incorporating these biomarkers are needed to identify high-risk patients and develop relevant treatment strategies. 

## 4. Triple-Negative Breast Cancer

Triple-negative breast cancer (TNBC) represents 15–20% of breast cancers. TNBC lacks ER, PR, and HER2 expression and is considered a heterogeneous and aggressive cancer [129,130]. TNBC is typically associated with poor prognosis and lacks targeted therapeutic strategies. Over the last few years, immunotherapy has gained traction in TNBC both in locally advanced and metastatic settings. Atezolizumab was the first immune checkpoint inhibitor approved in unresectable locally advanced or metastatic TNBC expressing ≥1% PD-L1. This approval was based on a phase III Impassion131 trial that, upon later review, was withdrawn by the FDA based on the lack of efficacy [131,132]. On 13 November 2020, the FDA granted accelerated approval to pembrolizumab in combination with chemotherapy in the treatment of unresectable locally advanced or metastatic TNBC breast cancer with a combined positive score (CPS) of ≥10. This approval was granted based on KEYNOTE-355 (NCT02819518), a multicenter, double-blind, randomized, placebo-controlled trial [133]. On July 26, 2021, the FDA approved pembrolizumab in locally advanced, high-risk TNBC for all comers regardless of PD-L1 expression. This approval was based on the results of the KEYNOTE-522 phase III trial [134]. Since these approvals, pembrolizumab has been the standard of care in TNBC but is also associated with considerable toxicities. Although the response rates have improved, there remains a need to identify patients who would benefit from immunotherapy, as 35% of patients do not achieve pCR [135]. Moreover, several patients experience toxicities and there is considerable financial toxicity with the use of immune checkpoint inhibitors. Identifying potential biomarkers to select patients benefiting from immunotherapy with the least toxicities is paramount. 

TNBC heterogenicity is determined by gene expression profiling, mutational copies, epigenetics, proteomics, and phospho-proteomics [136,137]. Lehmann et al. reported six molecular subtypes of the TNBC: basal-like 1 and 2 (BL1 and BL2), immunomodulatory (IM), mesenchymal (M), mesenchymal stem-like (MSL), and luminal androgen receptors (LARs) that were further classified into four subtypes (BL1, BL2, M, and LAR) [138,139]. The TNBC subtypes constitute different tumor microenvironments [TME], resulting in different immunotherapy responses. Molecular crosstalk between tumor inflammatory immune cells and immune modulatory cells plays a pivotal role in the tumor’s response to immunotherapy [129,140,141,142]. Although, along with PD-L1, as reported above, microsatellite instability (MSI-H) and high tumor mutational burden (H-TMB) are tumor agonistic markers approved for the use of checkpoint inhibitors in solid tumors, their use in TNBC is minimal due to the very low rate of MSI-H and H-TMB. Therefore, the PD-L1 expression in the form of CPA remained the primary biomarker in metastatic TNBC settings [143,144,145,146].

### 4.1. Molecular Basis of TNBC Heterogenicity

Lehman et al. reported BL1, BL2, M, and LAR as molecular subtypes in TNBC. These molecular subtypes result in variable therapeutic vulnerability to therapeutic agents [138,139]. Bareche et al. also reported TNBC molecular subtypes such as BL, M, LAR, MSL, and IM. They removed the BL2 subtype due to molecular instability [147]. Burnstein et al. also reported four distinct subtypes of the TNBC by combining gene expression profiling and copy number variations (CNVs). These four subtypes are LAR, mesenchymal (MES), basal-like immune-suppressed (BLIS), and basal-like immune-activated (BLIA) [136]. The IM and BLIA (basal-like immune activated)-related subtypes are characterized by a higher expression of immune gene signatures and targetable immune modulators, including immune checkpoints, and are associated with better prognosis [138,139,147,148]. The M and MSL tumors are associated with angiogenesis and the stroma signature, whereas the BL subtype is characterized by genomic instability, DNA gene repair deficiency, and a higher rate of TP53 mutation. The LAR subtype is characterized by the androgen receptor expression and is usually associated with a worse prognosis. Typically, the LAR subtype has higher incidences of CHH1, AKT1, and PIK3CA alterations [147]. 

Several studies have retrospectively evaluated the response to immunotherapy based on these molecular subtypes, revealing promising results. A retrospective analysis of the IMpassion130 trial in metastatic TNBC showed improved treatment-related outcomes in the BLIA subtype with atezolizumab, indicating the potential of immunotherapy in treating TNBC [149]. In another phase I trial (PCD4989), TNBC patients who received atezolizumab showed that the BLIA and LAR subtypes were associated with higher tumor-infiltrating immune cells such as TILs, PD-L1, and CD8-expressing immune cells compared to M and BLIS subtypes. In this study, BLIA and LAR subtypes were associated with better prognosis [150]. A 101-gene analysis based on different molecular classifications was reported in the NeoTRIPaPDL1 trial. In this trial, the pre-treatment TNBC subtype was not predictive of the response to the treatment. However, numerically, the pCR was higher (70%) in patients with BL1 tumors receiving atezolizumab and chemotherapy compared to 54% in chemotherapy alone. The pCR was low in both arms in LAR subtypes (22% vs. 19%). On the flip side, in patients with the M subtype of TNBC, the pCR was high (60% vs. 50%) in both arms [151,152]. 

**Figure 4:**
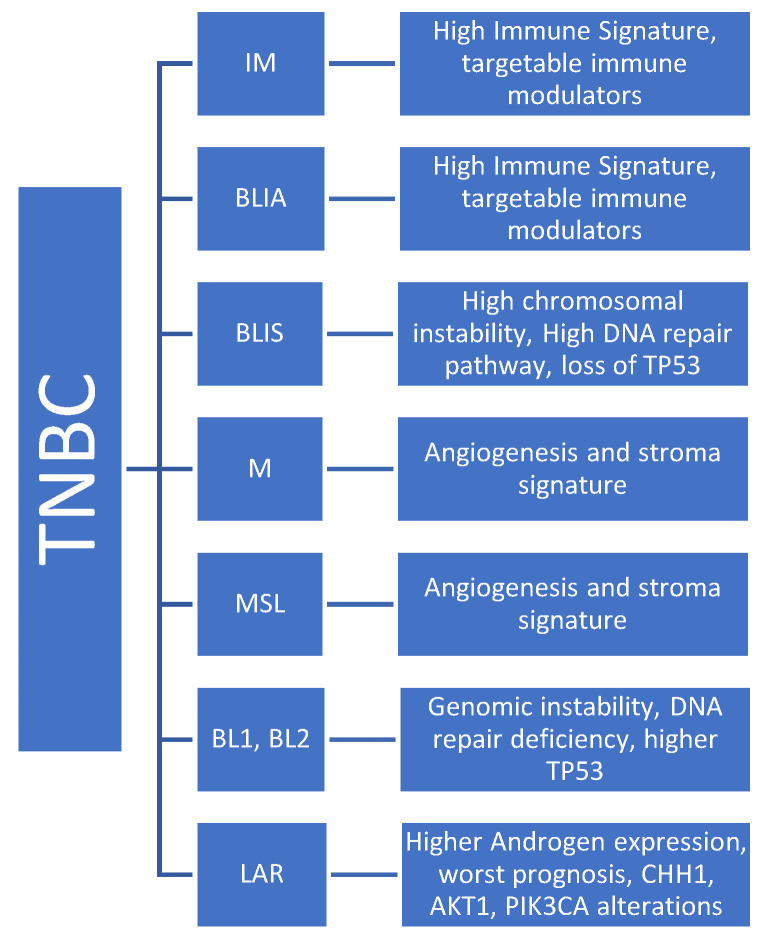
IM: immunomodulatory, BLIA: basal-like immune activated, M: mesenchymal, MSL: mesenchymal stem-like, BL: basal-like, LAR: luminal androgen receptor.

While these molecular subtypes can predict the response to immunotherapy, it is crucial to note that studies have shown molecular subtype evolution within the same patient, including changes in molecular subtypes with chemotherapy and/or immunotherapy. The most common change reported is the evolution from BL1 to M subtype in 38% of the cases following neoadjuvant therapy [153]. This underscores the need for further investigation into other prognostic biomarkers. However, clinical implications of molecular subtyping in routine practice have yet to be validated (Figure 4).

Biologic Subtypes of TNBC.

### 4.2. Programmed Death-Ligand 1 (PD-L1) Protein Expression as a Biomarker

PD-L1 is expressed in 15–50% of the TNBC. PD-L1 expression is higher in non-metastatic TNBC (up to 50%), whereas in metastatic TNBC, the expression is 15–20% [154,155]. That is why in locally advanced TNBC, immunotherapy is added to chemotherapy regardless of the CPS score compared to the metastatic TNBC, where CPR ³10 is mandated to combine immunotherapy with chemotherapy [134,135]. It has also been reported that immune-related molecular subtypes of TNBC, such as BLIA, M, and BL, show higher levels of PD-L1 expression (up to 78%), followed by BLIS (up to 32%), LAR (up to 35%), MES/MSL (up to 65%) [156,157,158]. Keynote 355 and Impassion 130 clinical trials demonstrated a predictive value of PD-L1 expression based on IHC [140,159]. However, TNBC patients respond regardless of the PD-L1 status, especially in locally advanced TNBC [134]. It has also been reported that the PD-L1 expression changes with the incorporation of other therapeutic modalities, such as chemotherapies, which can also change the responsiveness of tumors [151,160]. It is further observed that in Impassion 031 and KEYNOTE 522, the PD-L1 expression did not predict the pCR, and the pCR rate was consistent across all PD-L1 subclasses [161,162]. In the GeparNuevo trial, patients with higher PD-L1 expression receiving durvalumab in combination with chemotherapy demonstrated a higher pCR than the placebo arm [163]. However, overall, in current clinical practice, the utility of PD-L1 expression in locally advanced TNBC is experimental only. This difference in the predictive value of PD-L1 IHC expression between locally advanced non-metastatic and metastatic breast cancer could be related to the immune modulation and editing resulting from the immune suppression from TME in metastatic TNBC [164,165].

CD274 is a gene encoding the PD-L1 immune modulator. Molecular analysis of the CD274 gene amplification can provide a more accurate analysis of the PD-L1 expression as there is discordance between various IHC assays for the PD-L1 expression measurement. Each therapeutic agent has a companion diagnostic test in clinical utility [151]. In SAFIR02-BREAST IMMUNO, a phase 2 trial, PD-L1 assessment through a CGH array showed that a gain (3 to 4 copies) or an amplification (≥4 copies) of CD274 could predict the response to durvalumab in metastatic breast cancer [151]. 

### 4.3. Microsatellite Instability and Tumor Mutation Burden

The FDA approved MSI and TMB as tumor agonistic biomarkers for pembrolizumab use in solid metastatic or unresectable locally advanced cancers [143,144,145,146]. Higher neoantigen load within the tumor cells or TME leads to t-cell activation and tumor suppression. Higher TMB denotes a higher neoantigen load and acts as its surrogate. Higher TMB could lead to increased recruitment of the inflammatory cells into the TME and activate the adaptive immune response. This is mainly observed in TNBC compared to endocrine-positive tumors [145,166,167]. Although TNBC has higher immunogenic potential and a higher neoantigen load, the median TMB is still lower than the other solid tumors (1.8 mut/Mb) [146]. It has been reported that BL1 and M subtypes harbored more mutations than the different molecular subtypes. Moreover, TMB of >1.5 mut/Mb was associated with improved PFS [168]. The Keynote-158 trial, which led to the approval of pembrolizumab for solid tumors with TMB >10 based on the FoundationOne CDX assay, included 5–10% of TNBC patients [166,167]. However, this definition of higher TMB is controversial across different tumor types. In the NCT02091141 (MyPathway multi-basket) trial, a higher cut-off for TMB (≥16) was used. This trial demonstrated a higher response rate and an overall benefit from atezolizumab therapy across various tumor types regardless of MSI (38%). However, a limited efficacy was observed in patients with TMB of >10 but <16. This finding contradicts the findings of the Keynote-158 trial [169]. Pembrolizumab treatment improved CBR among patients with high TMB based on the exploratory analysis of Keynote-119 [145]. Similarly, in the GeparNuevo trial, high TMB (2.05 mut/Mb or higher) was associated with pCR with and without immunotherapy [170]. 

MSI results from the loss and gain of nucleotides in repetitive DNA microsatellite sequences. This increases tumor mutagenicity and neoantigen, resulting in heightened adaptive immunity [171,172]. Colorectal cancer and endometrial cancers have the highest rate of MSI. However, MSI is exceedingly rated in TNBC and is reported to have an incidence of 0.2% [172,173].

### 4.4. Tumor Infiltrating Lymphocytes

Tumor-infiltrating lymphocytes (TILs) constitute T-cells, B-cells, and natural killer (NK) cells. T-cells make up the higher proportion of the TILs [174]. The TIL proportion is higher in TNBC and, to an extent, HER2-amplified tumors than in luminal breast cancers [175]. The highest proportion of TILs is most seen in IM (38%) followed by BL2 (23%), MSL (21%), LAR (17%), BL1 (15%), and M (9%) molecular subtypes of the TNBC [139]. The International Immuno-oncology Biomarker Working Group on Breast Cancer has standardized the TIL scoring in breast cancer [176,177]. Integrating TIL biomarkers into the TNM American Joint Committee on Cancer (AJCC) Staging System for breast cancer is under consideration [178]. 

Higher TIL is reported to be associated with better clinical outcomes in localized and locally advanced/ metastatic breast cancers. In metastatic TNBC, higher levels of TILs are associated with improved ORR and OS [179]. TNBC patients with TIL scores of ≥10% responded better to atezolizumab plus nab-paclitaxel in the IMPassion-130 trial (HR: 0.64, 95% CI = 0.5–0.84) [159]. In early-stage breast cancer, a higher TIL level was associated with improved response to the treatment. In NeoTRIPaPDL1, a higher stromal TIL level was associated with higher pCR in response to the treatment with chemotherapy and atezolizumab [148]. In KEYNOTE-173 and GeparNuevo trials, the median increase in TILs from the baseline (both stromal and infiltrating TILs) before and after the treatment was associated with higher pCR as well, indicating the dynamic role of TILs during the treatment and its implications on the treatment-related outcomes [160,180]. TILs are used to identify TNBC with higher immunogenic potential and to choose therapy based on the immunogenic potential characterized by higher infiltrating TILs. In BELLINI, a phase II trial in the TNBC patients, nivolumab was administered with a low-dose ipilimumab or placebo. This trial met the biomarker-specific primary endpoint. In both cohorts, the increase in CD8+ T cells and/or interferon-gamma expression was 53% in the nivolumab cohort vs. 60% in the nivolumab/ipilimumab cohort. The TIL level was ≥40% in responders in both cohorts [181]. 

CD 8+ TILs are the most important immune cells influencing the response to immunotherapy. Multiple trials have shown a positive correlation between the expression of CD8+ T-cells and regulatory T-cells and response to immunotherapy/chemo-immunotherapy, such as Keynote 086, I-SPY2, and TONIC trials [182,183,184]. Further characterization of immune cells contexture within the TME has revealed three subtypes based on immunophenotyping in TNBC [185]. Fully inflamed phenotype (FI) is characterized by intra-tumoral localizations of TILs. A stroma-restricted (SR) phenotype is associated with the absence of infiltrating TILs but the presence of stromal TILs. The margin-restricted (MR) phenotype denotes the presence of TILs at tumor margins (Figure 2). Tumors with low TILs are also known as immune desert tumors (ID). The IM molecular subtype of TNBC has a higher proportion of FI tumors [142,156]. The M subtype has the lowest immunogenic potential as it has a higher prevalence of MR or ID phenotypes [139,142,156,168]. 

The immunogenic potential of the TNBC and the characterization of the TME are being explored further. However, further valuation studies are needed before this can be incorporated into clinical practice. As reported above, the integration of the TIL biomarker into the TNM American Joint Committee on Cancer (AJCC) Staging System for breast cancer is under consideration [178]. 

### 4.5. Immune Gene Expression in TNBC

RNA-seq of the tumor samples encompassing tumor cells and the TME. RNA-based expression profile tool can estimate the TME cells in tumor tissue and provide immune gene signatures, such as the MCP-counter, Cibersort) [186,187]. Immune gene expression in the TNBC and HER2-amplified tumors is reported to be associated with response to immunotherapy. The immune gene signature of breast cancer could reflect the immune cell population and the immunogenic potential of the tumor [188,189,190]. 

**Figure 5:**
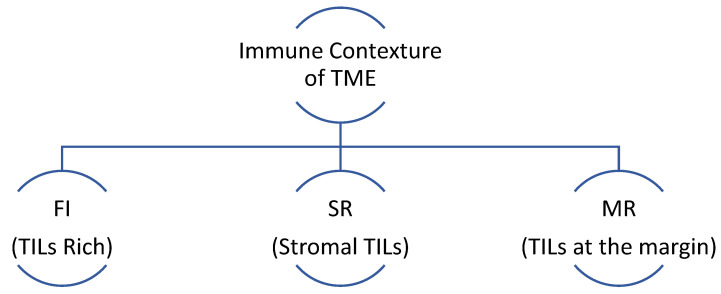
FI: fully inflamed has high intratumor localization of TILs, SR: stroma-restricted has the absence of infiltrating TILs but the presence of stromal TILs, MR: margin-restricted has TILs present at the margins only. TME: tumor microenvironment.

In TNBC, B and T cells gene expression signature resulted in better response to the pembrolizumab [150]. The tissue-resident memory (TRM), T cell signature, and 18-gene T cell-inflamed gene expression profile (GEP) were associated with better response to pembrolizumab [191]. Gene signatures indicating higher levels of STAT1 signature/chemokine 12 and dendritic cells were also associated with better response to immunotherapy (pembrolizumab) [192]. The GeparSixto immune gene expression signature (GSIS), TMB, and interferon signatures predicted the response to durvalumab therapy in the GeparNuevo phase II trial [170]. The GSIS signature provides 12 immune genes, dividing them into immune-cold and immune-hot genes. The genes are both immune-suppressive genes (*PDCD1*, coding for PD-1, *CD274*, coding for PD-L1, *CTLA4*, *FOXP3*, and *IDO1*) and immune-activating genes (*CCL5*, *CXCL9*, *CXCL13*, *CD80*, *CD21*, *CD8A*, *IGKC*) [179]. The NeoTRIPaPDL1 trial also demonstrated the predictive value of a 27-gene-based score and B-cell memory signature that can predict the response to atezolizumab in combination with chemotherapy [152]. Although these immune gene signatures offer hope for developing biomarkers that are more accurate in selecting patients with a higher likelihood of responding to therapy, these assays are costly, need special considerations, and suffer from a lack of standardization and validation on a larger scale (Figure 5) [193].

TME characterization by infiltrating immune cells.

## 5. Circulating Tumor DNA as a Predictive and Prognostic Biomarker

Circulating tumor DNA (ctDNA) consists of tumor-derived fragments of the DNA found in any body fluids such as blood/plasma, urine, cerebrospinal fluid, pleural fluid, and ascites. ctDNAs are typically encapsulated in the lipid membranes, trapped by the leukocytes, lipoproteins, or nucleosomes [194]. ctDNA was first described by Stroun et al. in 1987 and may contain driver and/or passenger mutations. It can potentially be a sensitive and specific cancer biomarker [195]. The National Cancer Institute further defines the term “Liquid Biopsy” as a test conducted on a sample of blood, urine, or other body fluids, as stated above, to look for cancer cells, DNA, or RNA pieces, or other molecules released by the tumor cells into the body fluids [196]. The non-invasive nature of the liquid biopsy is appealing for serial monitoring and detecting of the tumor fragments to inform the treatment decisions. Thierry et al. assessed the KRAS status via ctDNA in patients with metastatic colorectal cancer in 2014. This was one of the first studies reporting the clinical utility of the ctDNA in cancer patients. The cell-free DNA showed 100% sensitivity and specificity [197]. ctDNA monitoring has shown variable but promising results in several other tumor types [198,199,200]. 

Numerous techniques have been used to detect ctDNA with variable sensitivities. Highly sensitive techniques for ctDNA detection and characterization are based on NGS, digital droplet PCR (ddPCR), beads, emulsion, amplification, and magnetics (BEAMing) [201]. NGS is an ultrasensitive method to identify and quantify ctDNA at the lowest levels in the plasma. It depends on the protocols for low DNA input and designing biotinylated DNA oligonucleotide selectors. These selectors target the frequently mutated regions of the DNA specialized to the tumor type [202]. ddPCR is a powerful technique used to accurately quantify rare mutations. This technique relies on partitioning the samples into a multitude of units, with each unit containing one DNA molecule amplified by the PCR, resulting in amplification of the individual units and eventual detection [203]. BEAMing combines emulsion PCR with flow cytometry of the magnetic beads. Following amplification of the tumor DNA, the molecules are attached to the magnetic beads, and then further amplification occurs in numerous water-in-oil emulsion droplets. Each droplet contains a bead coated with DNA molecules labeled with fluorescence. Flow cytometry helps to identify and quantify these DNA molecules [204]. The bespoke assay is a tumor-informed assay that involves sequencing the tumor tissue and then identifying the tumor-specific genomic alterations in the blood with a sensitivity of 10–6 [205].

### 5.1. Role of ctDNA in Locally Advanced Early-Stage Breast Cancer

Detection of ctDNA in the blood can provide real-time monitoring of the tumor and inform therapeutic decisions. ctDNA and minimal residual disease (MRD) are used interchangeably. MRD represents cancer persisting after treatment, which is not seen by conventional imaging [206]. The MRD assessment in a curative intent setting in early-stage breast cancer can help plan adjuvant therapy. MRD at the end of curative intent therapy (e.g., surgery and/or adjuvant therapy) is associated with a risk of recurrence and impacts survival [207]. Approximately 85% of breast cancers are diagnosed at an early stage, and approximately 30% of these patients will relapse [208]. Several factors determine the risk of relapse after curative intent therapy, such as tumor stage, tumor size, nodal involvement, and histological grade [209]. 

Spot and longitudinal assessments of the ctDNA may provide early information regarding the risk of relapse and could be predictive and prognostic biomarkers following curative intent therapy [197,198,199,200]. The liquid biopsy detection of ctDNA can improve precision by detecting driver mutations. The ctDNA load is higher in progressive and metastatic settings. However, the ctDNA fraction is extremely low in early-stage breast cancers, where the detection of ctDNA requires highly sensitive methodology, such as a “Bespoke assay” that can detect the tumor-specific ctDNA down to the level of 10–6 [205]. CancerSEEK is another blood test that combines ctDNA and protein biomarkers, increasing the sensitivity of ctDNA detection to 43% in stage I, 73% in stage II, and 79% in stage III. This test has a specificity of 99% [210]. 

As mentioned above, longitudinal monitoring of the ctDNA can identify breast cancer patients with a higher risk of relapse. In a prospective study on 100 breast cancer patients, the primary tumors were sequenced for 14 driver mutations; 45 patients carry at least one of the driver mutations. The persistence of these driver mutations in ctDNA 2–4 weeks after the curative intent surgery was associated with the worst prognosis and resulted in earlier relapse [211]. It has been reported that serial monitoring of ctDNA can detect metastatic progression on an average of 11 months (0.5–37 months) before clinical, biochemical, and radiographic manifestations with 100% specificity and up to 93% sensitivity [212,213]. In another study, ctDNA provided a lead time of up to 3 years before the relapse could be evident from clinical and radiographic modalities [214]. 

Detection of MRD via ctDNA after curative intent therapy has excellent clinical utility. It provides better sensitivity and specificity with a lead time of up to 3 years. MRD assessment before and after adjuvant and neoadjuvant therapy provided an opportunity to modify treatment in high-risk patients. Poor prognostic factors, such as tumor stage, tumor size, nodal involvement, etc., are associated with higher ctDNA levels before neoadjuvant therapy. The ctDNA clearance is reported to be associated with higher pCR [215]. Another study reported that the patients clearing their ctDNA over neoadjuvant treatment compared to those who do not clear their ctDNA have better prognosis [216]. The C-TRAK TN study reported that 79% of TNBC patients with positive MRD developed metastatic disease. However, this study was skewed by the large proportion of high-risk patients [217]. In breast cancer, the utility of ctDNA and MRD testing is in evolution. More extensive studies are needed to validate the MRD assessment to direct the therapy in early-stage breast cancers. However, if MRD is proven to be an optimal surrogate marker for clinical trials, we can expect much faster clinical trials and endpoint reporting [208]. A negative MRD status can also be utilized to de-escalate the neoadjuvant and adjuvant therapy. This can have a substantial effect on mitigating the toxicity of cytotoxic neoadjuvant and adjuvant chemotherapy in breast cancer. In early-stage colon cancer, ctDNA-guided management spared the use of chemotherapy in about 15% of the patients, and the treatment-related outcomes were similar in patients with and without chemotherapy [218].

### 5.2. Role of ctDNA in Metastatic Breast Cancer

ctDNA in metastatic cancer can have several clinical utilities. It can be a prognostic marker, informative of the tumor burden, and a monitoring tool. It can also inform the genetic alterations and clonal evolutions in the ctDNA that can be potential therapeutic targets [219,220]. ctDNA has high sensitivity in metastatic breast cancer and can detect tumor-derived mutations in up to 85.7% of patients compared to 57.8% in stage I-III patients [221]. 

In active metastatic disease, ctDNA levels are high and could be used to monitor the disease evolution, predict response to the treatment, and act as prognostic tools, as a higher level of ctDNA is associated with poor survival. ctDNA percentage is quantitatively associated with the clinical outcomes, as higher ctDNA levels are associated with shorter OS [212,222,223]. The LOTUS and INSPIRE phase II trials also reported that the ctDNA levels correlate with the overall clinical response, PFS, and OS [224,225]. Moreover, ctDNA in metastatic settings can have a lead time of several months before clinical and radiographic progression is observed, providing a more personalized approach and the potential to change therapy early on in the course [224]. 

ctDNA is now used to detect emerging mutations and clonal evolution during the treatment. This has limited the utility of tissue biopsies [219,226]. There is an increasing concordance between the tissue biopsy and the ctDNA-based molecular analysis due to more sensitive techniques for ctDNA detection. Per the plasma MATCH study, 98% of the mutations detected by ctDNA coincided with the detection by tissue biopsy [227]. Moreover, ctDNA can provide tumor heterogenicity more accurately than tissue biopsy due to the lack of uniform tumor tissue and the potential for inaccessible lesions for tissue biopsy [228]. 

ctDNA can detect several mutations in breast cancer that can be the targets for multiple therapeutic agents. The most common mutations that ctDNA detects in breast cancer are TP53, PIK3CA, ESR1, GATA3, PTEN, and ARID1A [229]. Although these mutations provide therapeutic targets, typically, they are used after progression on prior lines of therapy. Currently, ongoing trials are investigating the incorporation of these therapeutic agents at the appearance of targeted mutation before clinical and radiographic evidence. Currently, SERENA-6, a phase 3 trial, is investigating the early incorporation of ESR-1-directed therapy in detecting ESR-1 mutation in patients with stable disease on CDK4/6i plus AI. The patients are randomized to continue the same treatment or switch AI to an oral SERM. The primary endpoint of this trial is PFS [230]. In another phase 2 trial (PADA-1 trial), improvement in PFS was observed by switching from AI + palbociclib to fulvestrant + palbociclib after the appearance of ESR1 mutation [41]. However, in both trials, the primary endpoint was PFS, which may not be as reliable as OS. Trials with more optimal endpoints will be needed to see early switch therapy’s impact before clinical and radiographic progression. Several other mutations with potential targets or clinical applications are being detected by the ctDNA, as enlisted in Table 2. 

**Table 2:** Molecular targets and therapeutic implications.

Gene Mutation	Clinical Utility
ESR1 Hotspots: Y537C, Y537N, Y537C, S463P, D538G	Resistance to the endocrine therapy. Therapeutic agents: elacestrant, camizestrant, imlunestrant, lasofoxifene, rintodestrant [47,48,49,50,51,52,53,54,55,56,57,58,59,60,61],
AKT Hotspots: E17K	Therapeutic agent: capivasertib, ipatasertib (AKT kinase) [227,231].
HER2 Hotspots: V777L, L755S	Increased sensitivity to HER2 targeted therapies. Therapeutic targets: neratinib, lapatinib (bind to kinase domains) [232,233,234]
PTEN Hotspots: R130Q, R130G, R130*, R130P, R130Qfs*4	Confers resistance to PI3Ki (loss of PTEN) and confers sensitivity to AKT inhibitors. Therapeutic targets: capivasertib, ipatasertib (AKT kinase) [227]
PIK3CA Hotspots: H1047R, H1047L, N345K, E545K, E524K, E726K	Truncal mutation confers resistance to endocrine therapy. Therapeutic agents: alpelisib, taselisib, buparlisib, copanlisib, capivasertib [227,228,229]

ESR1: estrogen receptor 1, AKT: protein kinase B, human epidermal growth factor receptor 2, PTEN: phosphatase and tensin homolog deleted on chromosome ten, PIK3CA: phosphatidylinositol-4,5-bisphosphate 3-kinase.

Overall, ctDNA is a powerful tool that is emerging as a great clinical tool for therapeutic selection and a predictive and prognostic marker. The evidence on the utility of ctDNA as a monitoring and therapeutic selection tool is evolving, but more clinical trials are needed before it could become a norm in breast cancer. 

## 6. Conclusions

Breast cancer is a heterogeneous malignancy with several biological and molecular subtypes. The treatment of each type of breast cancer differs significantly, with considerable overlap. Several pathways drive these subtypes. Promising therapies are emerging as more of these pathways are better understood. The activation/inactivation and amplification of these pathways result in cancer growth and escape.

Moreover, identifying these pathways has paved the way for recognizing potential diagnostic, prognostic, and predictive biomarkers such as PDL-1 in TNBC. Moreover, the immune constitution of TME, molecular cross-talk, and application of modern technologies such as transcriptomics, proteomics, and individual cell sequencing can lead to better development of therapeutics and novel biomarkers. Although hormone-positive, TNBC, and HER2-positive breast cancers are biologically distinct, there are some common therapeutic agents (besides traditional chemotherapy) used in each of these subtypes. One example is fam-trastuzumab-deruxtecan-nxki, which has been approved in hormone-positive, TNBC (HER2 low, HER2 IHC 1+, 2+) and HER2 breast cancers in metastatic settings based on Destiny trials [235,236]. Sacituzumab govitecan-hziy is another therapeutic agent approved for metastatic hormone-positive breast cancer and TNBC [237,238]. Pembrolizumab is approved in a tumor-agonistic fashion in cancers with a high tumor mutation burden [239]. These indicate common molecular pathways involved in the pathogenesis of a number of malignancies, including subtypes of breast cancer.

Overall, precision medicine has revolutionized cancer diagnostics and therapeutics in general, particularly in breast cancer. Cancer treatments are more tailored and have minimized toxicities without compromising much on efficacy. Patients with breast cancer are living longer as novel therapeutics are emerging. However, predictive and prognostic biomarkers, immune response monitoring, and potential biomarkers for toxicity assessment remain areas of need. 

## Data Availability

The raw data supporting the conclusions of this article will be made available by the authors on request.
